# Case Report: Reversal of Hyaluronic Acid Rectal Wall Infiltration with Hyaluronidase

**DOI:** 10.3389/fonc.2022.870388

**Published:** 2022-04-26

**Authors:** Anne Hong, Joseph Ischia, Michael Chao

**Affiliations:** ^1^ Department of Urology, Austin Health, Melbourne, VIC, Australia; ^2^ Department of Radiation Oncology, Austin Health, Melbourne, VIC, Australia

**Keywords:** hyaluronic acid, hyaluronidase, prostate cancer, radiation therapy, rectal spacer

## Abstract

Peri-rectal spacers provide protection to the rectum for patients receiving radiation therapy treating prostate cancers. Commonly used hydrogel spacers hold the disadvantage that they cannot be readily reversed should inadvertent injection outside of the target area occurs, potentially leading to ischemia of the rectal mucosa leading to severe pain and ulceration, which can then lead to superinfection and pelvic abscess formation, and subsequently recto-prostatic fistulas. This could require major surgical intervention. New hyaluronic acid spacers are readily reversible with hyaluronidase and provide a valuable means to correct any misinjected spacer. We present a patient with prostate cancer who was planned for radiation therapy and required a rectal spacer. The hyaluronic acid rectal spacer was injected in part into the rectal wall. The patient was asymptomatic, and a sigmoidoscopy confirms healthy bowel mucosa only. The misinjected hyaluronic acid was successfully treated with targeted injection of hyaluronidase into only the rectal wall portion. Serial follow-up imaging demonstrated rapid dissolution of the misinjected hyaluronic acid with the well-positioned hyaluronic acid remaining. The patient did not experience any side effects of the hyaluronidase.

## Introduction

Peri-rectal spacers have been shown to be effective in reducing toxicities resulting from radiation therapy for prostate cancer (1). The goal of rectal spacing is to position the rectal wall temporarily away from the prostate, keeping it safely distant from the high-dose region. The spacer is implanted using a transperineal approach under trans-rectal ultrasound (TRUS) guidance, together with the placement of fiducial markers in the prostate. In rare occasions, it is possible to inadvertently puncture part of the rectal wall, which may cause misplacement of a portion of the implant. This could cause potential ischemia to the rectal wall, ultimately leading to severe complications if left unnoticed.

For spacers composed of hyaluronic acid (HA), the resorption process naturally occurs slowly *via* the enzyme hyaluronidase (HAS). In dermal applications, HA implants (fillers) are reversed more quickly by injecting exogenous HAS (2). This is the first known case of reversing a HA peri-rectal implant with the use of HAS.

## Case Presentation

The patient was a 69-year-old man with a newly diagnosed Gleason 4 + 4 = 8 prostate cancer who was scheduled for high-dose intensity modulated radiation therapy (IMRT). A prostate-specific membrane antigen positron emission topography (PSMA PET) scan showed localized disease. The prostate and seminal vesicles were planned to receive hypofractionated radiation therapy to a dose of 60 Gy in 20 fractions. The patient was started on androgen deprivation therapy with leuprorelin. In further preparation for his treatment, three gold fiducial markers were implanted into his prostate under a general anesthetic for image-guided radiation therapy. Additionally, a rectal spacer (Barrigel^®^, Palette Life Sciences, Santa Barbara, California, USA) was also implanted into the peri-rectal fat between Denonvillier’s fascia and the anterior rectal wall. The goal of the implant was to create approximately 1 cm of symmetrical separation between the prostate and rectal wall, from the base to the apex of the prostate.

**Figure 1:**
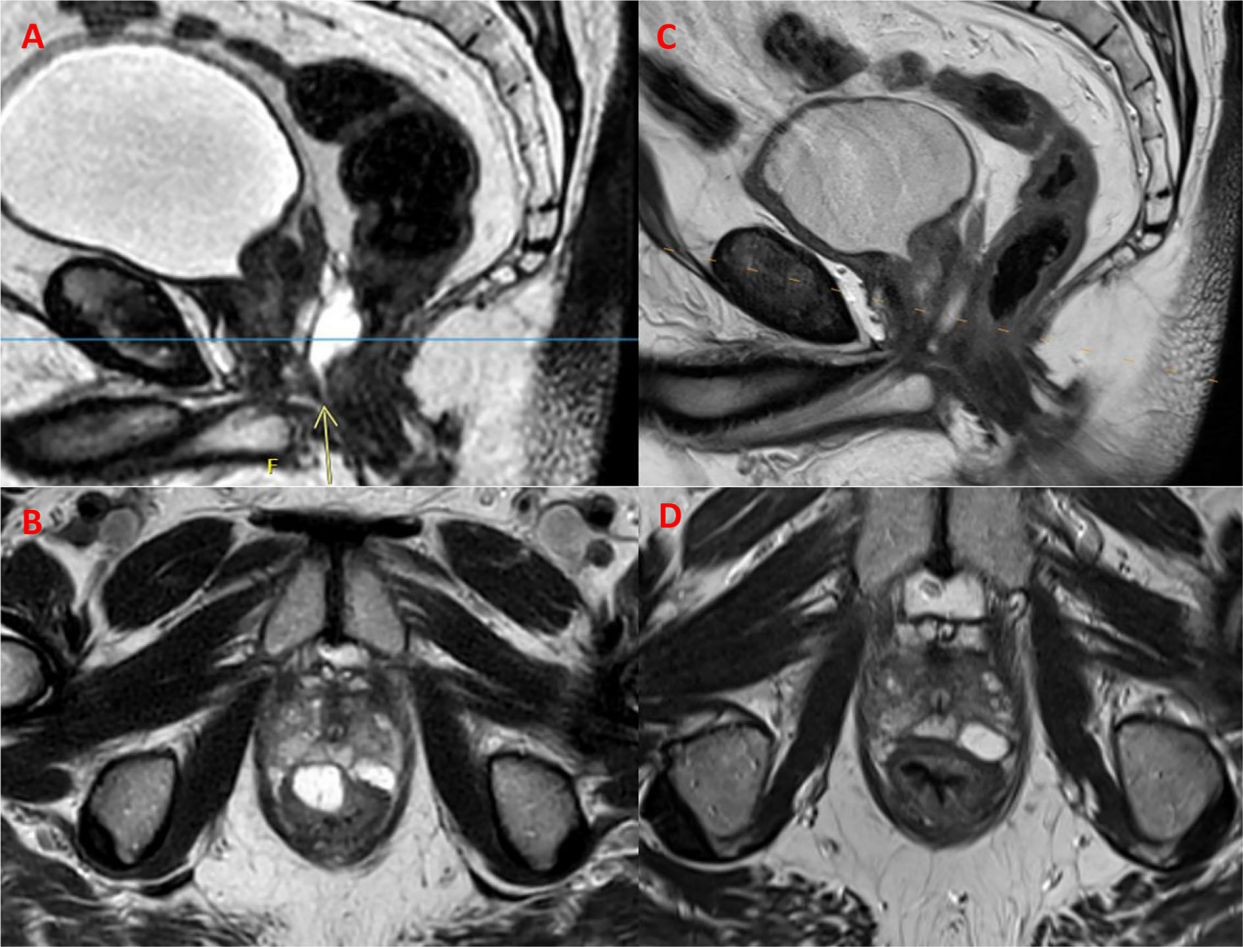
Magnetic resonance images demonstrating intramural non-animal stabilized hyaluronic acid (NASHA) in **(A)** sagittal and **(B)** axial views denoted by*. Two days post hyaluronidase injection, the intramural NASHA is no longer visible in the same views **(C, D)**.

The technique involved the use of a midline 18G needle inserted transperineally into the perirectal fat using a freehand approach under sagittal TRUS guidance. The patient was observed to have a large rectal hump (arising from the rectourethralis muscle), which required a challenging angle of entry for the needle into the perirectal fat. Approximately 9 cc of HA was inserted along a length of 4.5 cm extending from the base to the apex of the prostate. Upon routine review of the post-implant MRI images 2 weeks after HA insertion, a portion of the HA implant at the level of the prostate apex was determined to have infiltrated the rectal wall into the muscularis propria layer (**Figure 1**). **Figure 1B** shows the low-density anterior border of the misinjected HA, with the low-density area corresponding to the muscularis propria layer of the rectal wall. This volume of HA was estimated at 5 cc out of a total of 10 cc of HA delineated on MRI. It extended from the midline to the right of the prostate for 2 cm, starting at the apex and extending superiorly for 3 cm. Using the grading scale initially described by Fischer-Valuck et al., our case would constitute grade 3 rectal wall infiltration (3). The needle had inadvertently penetrated the rectal wall during its entry into the perirectal fat at the level of the rectal hump, and as the needle was withdrawn while injecting HA, this resulted in a portion of HA in the intramural location. The patient was asymptomatic, denying any pain, bleeding, or tenesmus. A sigmoidoscopy was performed, which confirmed an intact rectal mucosa. As a significant portion of the HA was determined to be within the rectal wall, the patient’s IMRT was withheld to prevent any potential spacer-related toxicity. However, to avoid any prolonged delays with commencement of his IMRT, a decision was made to dissolve the portion of HA that had infiltrated the rectal wall.

**Figure 2:**
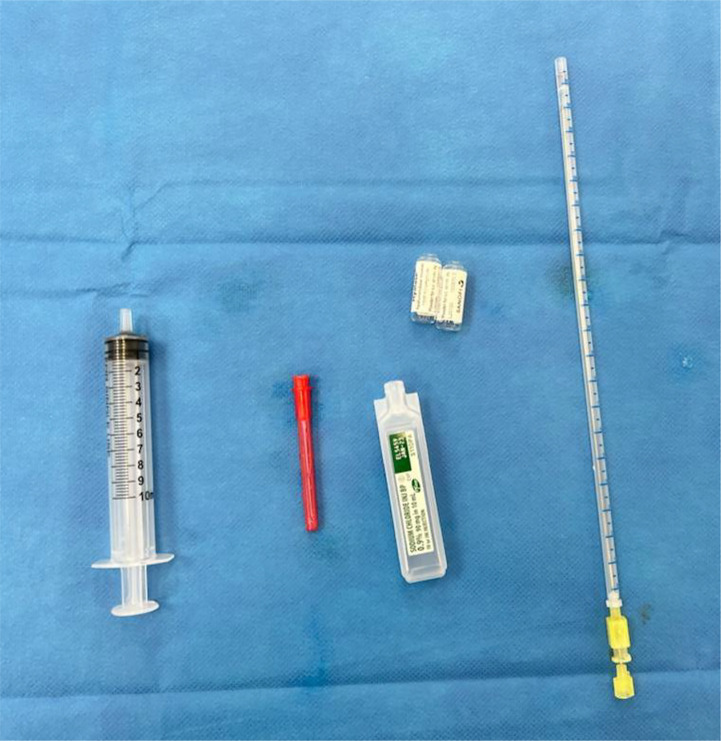
Equipment required for preparing hyaluronidase (left to right)—10 cc syringe, blunt drawing needle, normal saline, hyaluronidase, and spinal needle.

A sub-dermal patch test was done on the patient before his GA to check for any rare allergic reaction. Twenty units of HAS was injected intra-dermally in the forearm with a 25 G needle, and after 30 min, the injection site was assessed for any weal, itching, or erythema. No reaction was observed. As we had planned to dissolve 5 cc of HA within the rectal wall, at least 30 U of HAS per 0.1 cc of HA was required (4). HAS (3000 U) in 6 ml of sterile saline was prepared for injection. The equipment used is shown in **Figure 2**.

**Figure 3:**
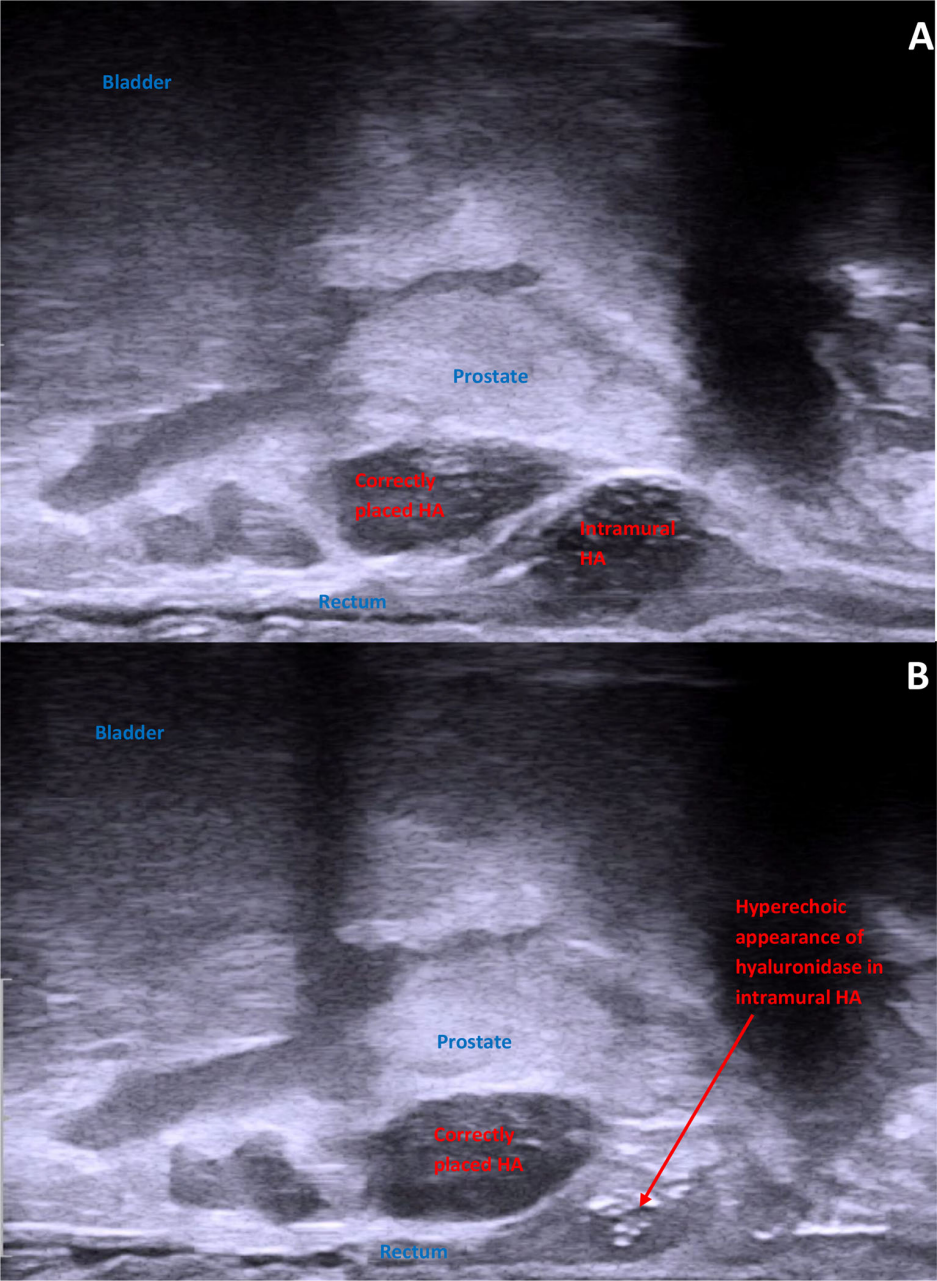
Intraoperative transrectal ultrasound images demonstrating **(A)** the misinjected HA in the rectal wall and **(B)** the hyaluronic acid immediately after injection into the intramural NASHA (now hyperechoic).

The patient underwent general anesthesia and was positioned as for the original HA procedure and imaged with TRUS confirming the location of the HA within the rectal wall (**Figure 3**). The HA was clearly visible on TRUS. A 20 G Chiba biopsy needle was inserted transperineally using a freehand approach into the middle of the HA bleb and advanced towards its superior extent. HAS (2000 IU) was injected into the HA bleb as the needle was withdrawn to its inferior extent. The HA bleb became hyperechoic on TRUS after the injection. No immediate dissolution was noted (**Figure 3B**). We were also unable to extract any part of the HA bleb by aspirating with an 18 G rigid BP needle. The entire procedure lasted approximately 20 min and was considered straightforward.

During follow-up, the patient remained asymptomatic immediately post procedure, at day 2, day 7, and day 14. MRI scans on day 2, day 7, and day 14 post HAS injection demonstrated complete reabsorption of the intramural HA at day 2 (**Figure 1**). There was some reabsorption of the HA within the peri-rectal fat at day 2 but only minimal additional reabsorption was seen at day 7. By day 14, no further ongoing changes were seen. The patient has since been scheduled to undergo radiation therapy planning with the goal of resuming his planned IMRT within 4 weeks. There were no changes to the dose of IMRT planned.

## Discussion

Prior to the development of HA, hydrogels and balloons were available for use as rectal spacers in prostate cancer. Balloon spacers may significantly reduce the radiation dose to the rectum (5) but have been associated with rectal perforation (6, 7). This in turn may lead to delays in radiation for the primary issue of prostate cancer (7) and require further intervention. Hydrogel spacers sustained less volume loss throughout the treatment period (5); however, they cannot be reversed and need to be surgically removed if they were infiltrated into the rectal wall.

HA was first approved for use as a cosmetic filler by the United States’ Food and Drug Administration (FDA) in 2003, and since then, it has experienced exponential growth in popularity and use (2). A unique property of HA, including HA that is misplaced or overfilled, is that it can be reversed with HAS (8). This ability to reverse and remodel hyaluronic acid is advantageous as it allows correction of any inaccurately placed product and can minimise adverse events, as in our case. Other rectal spacers such as hydrogel cannot be reversed and would need to be surgically removed. Some degree of rectal wall infiltration was found to occur in 6% of cases in the randomized hydrogel spacer trial (3), none of which required further intervention. However, the potential consequences of more severe gross rectal wall infiltration may include ischemia of the rectal mucosa leading to severe pain and ulceration, which can then lead to superinfection and pelvic abscess formation, and subsequently recto-prostatic fistulas requiring major surgical intervention such as a defunctioning ileostomy/colostomy or even pelvic exenteration (9, 10). Notably, McLaughlin et al. described a patient receiving high-dose stereotactic body radiation therapy. The radiation dose may have contributed to the formation of a rectourethral fistula ultimately managed with pelvic exenteration (10). Nevertheless, we have now demonstrated that this risk can be mitigated by early recognition and the use of HAS to rapidly reverse portions of the implant, preventing subsequent downstream severe complications.

Although HAS has been used routinely to dissolve dermal and breast HA fillers (see below), this is the first report of the use of HAS in reversing a peri-rectal HA implant. The dose of HAS recommended has ranged between 5 and 30 IU for every 0.1 cc of HA to be dissolved. At these doses, multiple HAS injections may be necessary to completely dissolve any undesired HA. As such, we decided to increase the dose, beyond the upper end of the recommended dose of HAS in order to ensure we did not require a second procedure. In addition, there is no known upper limit for the amount of HAS that can be injected safely. While the maximal dose of HAS is not documented, up to 200,000 IU has been given, demonstrating an increase in allergic-type reactions (11). Our patient received well under this dose, and thus the risks of adverse reactions are minimized. We used approximately 50 IU per 0.1 cc of HA and saw rapid dissolution of the HA that had infiltrated the rectal wall. This had quickly dissolved within 48 h. If the patient had been symptomatic with severe pain, this would have resulted in rapid resolution of his symptoms. In addition, it would have averted potentially prolonged clinical symptoms as it may take 1 year for HA to resolve naturally. This procedure also allowed us to reschedule the start of the patient’s IMRT with minimal delay.

Additionally, HA is clearly visualized on several imaging modalities including ultrasonography and MRI, and to a lesser extent computer tomography. This allows clinicians to precisely assess the position and volume of any inaccurately placed HA and can help facilitate the calculation of the HAS dose required. During HAS injection, visualization under ultrasound can guide the accurate placement of HAS. Using this guidance, we have shown that it is possible to target only the misplaced portion of the implant with HAS, while leaving the remaining portion of the implant in the correct position.

Complications of HAS in the cosmetic surgery setting are well documented. These include allergic reactions, which ranges from 0.05% to 0.69% in frequency; the majority of these are reported to be localized injection site reactions (12). Systemic reactions such as angioedema and urticaria can occur at a lower frequency (<0.1%). Higher dose (more than 100,000 IU) and intravenous route of administration are more likely to produce allergic reactions (12). Specific to urology, the injecting needle could injure the surrounding organs. Inadvertent placement of HAS into the correctly placed HA could lead to over-dissolution of the rectal spacer leading to increased toxicity from their IMRT due to loss of the protective rectal spacer. However, once adequate time has passed, there is the potential to insert additional HA into the peri-rectal space if this was deemed important.

Several learning points arise from our case. During transrectal ultrasonography with the sagittal view, the rectal wall is tented by the rectourethralis muscle near the apex of the prostate. When injecting HA, the needle should always pass above rather than through the rectal wall to minimize the risk of injury to the rectal wall. In addition, HA should always be inserted when the needle tip is in clear view during the entire procedure. As HA does not polymerize, there is no time constraint with the insertion process. HA is also clearly visible on TRUS imaging, and it does not distort or degrade the rectal or prostate images, allowing us to accurately track the insertion to minimize the risk of rectal wall infiltration. We would also recommend performing an MRI scan to help delineate the location of the HA and identify any patients who may have gross rectal wall infiltration. This would be difficult to identify on a CT scan. Furthermore, onset of action of HAS is within minutes and effects last up to 48 h (4, 11). Therefore, it is expected that patients with painful symptoms due to rectal wall infiltration could experience rapid relief after HAS injection. A repeat MRI can be performed at 2 days post HAS injection to demonstrate the resolution of the HA. No further changes were noted at 2 weeks post HAS injection, allowing us to repeat the planning images for radiation therapy. This translates to a small and clinically insignificant delay in initiation of their IMRT as opposed to many months delay in the case of non-reversible preparations.

## Conclusion

HA use as a rectal spacer is safe and can reduce the toxicity of radiation therapy to the prostate. In the event of rectal wall infiltration, HA can be simply and readily reversed with HAS.

## Data Availability Statement

The raw data supporting the conclusions of this article will be made available by the authors, without undue reservation.

## Ethics Statement

Ethical review and approval were not required for the study on human participants in accordance with the local legislation and institutional requirements. The patients/participants provided their written informed consent to participate in this study. Written informed consent was obtained from the individual(s) for the publication of any potentially identifiable images or data included in this article.

## Author Contributions

JI was the treating clinician of the patient. JI and MC provided supervision for this body of work. AH drafted the manuscript that was edited by all authors. All authors contributed to the article and approved the submitted version.

## Conflict of Interest

MC is an advisory board member for Palette Life Sciences Pty Ltd.

The remaining authors declare that the research was conducted in the absence of any commercial or financial relationships that could be construed as a potential conflict of interest.

## Publisher’s Note

All claims expressed in this article are solely those of the authors and do not necessarily represent those of their affiliated organizations, or those of the publisher, the editors and the reviewers. Any product that may be evaluated in this article, or claim that may be made by its manufacturer, is not guaranteed or endorsed by the publisher.
